# *Acinetobacter lwoffii* Peritonitis in a Patient on Automated Peritoneal Dialysis: A Case Report and Review of the Literature

**DOI:** 10.1155/2017/5760254

**Published:** 2017-07-26

**Authors:** Murat Yasar Tas, Meral Merve Oguz, Mevlut Ceri

**Affiliations:** ^1^Department of Nephrology, Faculty of Medicine, Pamukkale University, Denizli, Turkey; ^2^Department of Internal Medicine, Faculty of Medicine, Pamukkale University, Denizli, Turkey

## Abstract

*Acinetobacter lwoffii*, a nonfermentative gram-negative aerobic bacillus, which presents in the normal flora of the oropharynx and skin, has recently been reported as a cause of human infection. Herein, the authors present a case report of peritonitis related to automated peritoneal dialysis caused by* A. lwoffii.*

## 1. Introduction

Peritonitis is a life threatening condition of peritoneal dialysis (PD), and gram-negative peritonitis ratio has recently been increased. Gram-negative peritonitis, which is associated with a higher risk of hospitalization and death, is clinically more severe [1, 2]. Herein, we present a peritonitis caused by* A. lwoffii* in a diabetic patient on automated peritoneal dialysis (APD).

## 2. Case Presentation

A 42-year-old man receiving APD therapy for approximately one year was admitted to the hospital with diffuse abdominal pain and cloudy dialysate for two days. His medical history included diabetes mellitus (DM) type 2, hypertension, and coronary heart disease but no episodes of peritonitis. He was smoking for twenty years. Physical examination was normal without hypotension, fever, and diffuse abdominal tenderness, and no finding of infection was observed at the catheter exit site or tunnel. His laboratory tests were as follows: hemoglobin: 10.4 g/dL; white blood cell count (WBC): 6390/mm3 (with 76% neutrophils); BUN: 47 mg/dL; creatinine: 5,55 mg/dL; albumin: 3.8 gr/dL; erythrocyte sedimentation rate: 102 mm/h; C-reactive protein (CRP): 22 mg/dL; and peritoneal fluid WBC: 10.380 cells/mm3 (with 90% neutrophils). Gram stain was negative and standard dialysate culture technique was performed. Ceftazidime 1 × 1 g intraperitoneal and piperacillin/tazobactam 3 × 2,25 g intravenous empiric treatment had been started because of the patient's septic condition. On the third day of the treatment, peritoneal fluid and blood cultures identified* A. lwoffii* that was sensitive to many antibiotics. Clinical and laboratory improvement (peritoneal fluid WBC 300/mm3 and CRP 13 mg/dL) were prominently observed on the third day of treatment; therefore antibiotic treatment was exactly continued and the control dialysate culture was sterile. He was given antibiotic treatment for 15 days to cure the peritonitis and discharged without any problems.

## 3. Discussion

*Acinetobacter lwoffii* is a nonfermentative gram-negative aerobic bacillus. This bacterium presents in the normal flora of the oropharynx and skin, which is an important pathogen in nosocomial infections and immunocompromised patients [3]. However, pneumonia, acute gastroenteritis, liver abscess, septicaemia, and endocarditis are reported as cases of community-acquired infections related to* A. lwoffii * [3–7]. In literature, PD-related peritonitis caused by* A. lwoffii* has rarely been reported [2, 8, 9]. Huddam et al. documented the fact that community-acquired* A. lwoffii* peritonitis was successfully treated with early recognition and appropriate antibiotic therapy. On the other hand, one patient with previous* A. lwoffii* peritonitis had been documented in a review of seven* A. baumannii* peritonitis cases [8]. Zhang et al. retrospectively analyzed 26 episodes of PD-related* Acinetobacter* species peritonitis and nine peritonitis episodes associated with* A. lwoffii* were identified [9].

*Acinetobacter* species infections tend to occur in patients with chronic diseases as diabetes mellitus, chronic obstructive pulmonary disease, renal disease, heavy smoking, and excess alcohol consumption [2]. Chao et al. stated that DM and chronic glomerulonephritis are the most common causes of renal disease in the* Acinetobacter* peritonitis patients [9]. Similarly, our patient had comorbidity as DM, renal disease, and smoking.

Galvao et al. proposed that* Acinetobacter* peritonitis tends to occur within the first 1-2 months following another PD peritonitis episode [2, 10]. In contrast, Chao et al. showed that most* Acinetobacter* peritonitis did not follow the previous peritonitis after the year of 2000, but there were on average 2 episodes with different pathogen before the* Acinetobacter* peritonitis [9]. However, there was no preceding peritonitis caused by a different pathogen in our case and this was the patient's first peritonitis episode.

It has been reported that* A. baumannii *may escape the effects of conventional antimicrobial therapy [1]. In contrast, the other* Acinetobacter* species than* A. baumannii* are highly susceptible to ciprofloxacin, ampicillin/sulbactam, gentamicin, tigecycline, and carbapenem [11]. In reviewing literature,* Acinetobacter* peritonitis was usually treated with early and appropriate antibiotic therapy instead of catheter removal [2, 9]. Similarly,* A. lwoffii* was sensitive to many antibiotics in our case's antibiogram and we treated the patient without necessary Tenckhoff catheter removal.

There are several therapeutic options for the treatment of antibiotic-susceptible* Acinetobacter *infections. These are cephalosporin (ceftazidime or cefepime), a combination of beta-lactam/beta-lactamase inhibitor, or a carbapenem (imipenem, meropenem, or doripenem). For the resistant isolates, therapeutic options are polymyxins and tigecycline. We continued treatment with piperacillin/tazobactam due to good response to treatment. According to our best knowledge, there is no clue for the optimal treatment period for* A. lwoffii *peritonitis.

In conclusion,* A. lwoffii* has increasingly been recognized as an important pathogen in PD-related peritonitis which can be treated successfully with appropriate antibiotic treatment instead of catheter removal.
